# Efficacy of different rehabilitation therapies on post-stroke aphasia patients: A network meta-analysis

**DOI:** 10.1097/MD.0000000000038255

**Published:** 2024-05-24

**Authors:** Congli Han, Jienuo Pan, Jinchao Du, Luye Feng, Hengqin Ma, Jiqin Tang

**Affiliations:** a College of Rehabilitation Medicine, Shandong University of Traditional Chinese Medicine, Jinan, Shandong, China; b College of Rehabilitation, Weifang Medical University, Weifang, Shandong, China; c The First Clinical Medical College of Shandong University of Traditional Chinese Medicine, Jinan, Shandong, China.

**Keywords:** aphasia, network meta-analysis, randomized controlled trial, rehabilitation intervention

## Abstract

**Background:**

Although several rehabilitation interventions are effective in post-stroke aphasia (PSA), the efficacy of different rehabilitation interventions compared to each other remains controversial. Here, we aimed to compare the effectiveness of varying rehabilitation interventions in PSA.

**Methods:**

Randomized controlled trials on 8 kinds of rehabilitation interventions to improve speech function in patients with PSA were searched by computer from 10 databases, including PubMed, Web of Science, Cochrane, OVID, CINAHL, Embase, CNKI, WanFang, CBM, and VIP. The search scope was from the establishment of the database to August 2023. The literature screening, extraction of basic information, and quality assessment of the literature were conducted independently by 2 researchers. Network meta-analysis (NMA) was performed using Stata 17.0 software.

**Results:**

Fifty-four studies involving 2688 patients with PSA were included. The results of NMA showed that: ① in terms of improving the severity of aphasia, the therapeutic effects of repetitive transcranial magnetic stimulation were the most significant; ② motor imagery therapy was the most effective in improving spontaneous speech, repetition, and naming ability; ③ in terms of improving listening comprehension ability, the therapeutic effects of mirror neuron therapy was the most significant.

**Conclusion:**

The 8 rehabilitation interventions have different focuses in improving the speech function of PSA patients, and the clinical therapists can select the optimal rehabilitation interventions in a targeted manner according to the results of this NMA and the patients’ conditions and other relevant factors.

## 1. Introduction

Post-stroke aphasia (PSA) is an acquired language disorder caused by damage to the dominant hemispheric speech area as a result of stroke.^[1]^ It is characterized by multifaceted language dysfunction in phonology, morphology, semantics, and syntax.^[2]^ Research shows that about 32% of post-stroke survivors have different degrees of speech disorders, and about 60% of patients still have such symptoms 1 year after the onset of stroke, which seriously affects their daily communication and social participation and hinders their rehabilitation process.^[3,4]^ Failure to provide timely and correct rehabilitation interventions may lead to psychological disorders, which may have a negative impact on the family and society.

In recent years, traditional speech rehabilitation therapies such as Schuell stimulation therapy (SST), music therapy (MT), and constraint-induced language therapy (CILT) have been widely recognized in the clinical treatment of PSA.^[5,6]^ Meanwhile, emerging rehabilitation interventions such as attention training (AT), motor imagery therapy (MIT), mirror neuron therapy (MNT), repetitive transcranial magnetic stimulation (rTMS), and transcranial direct current stimulation (tDCS) have been successively used in the treatment of PSA patients, and have also shown sound therapeutic effects.^[7–9]^ However, the efficacy of different rehabilitation interventions compared to each other remains controversial. Network meta-analysis (NMA) allows for direct and indirect comparisons of multiple rehabilitation interventions simultaneously and analyzes the clinical effectiveness of more than 2 therapies. Thus, this study systematically evaluates 8 rehabilitation interventions commonly used to treat PSA through NMA. It compares the intervention effects of each rehabilitation therapy to provide a reference basis for selecting rehabilitation intervention methods for the clinical treatment of PSA.

## 2. Methods

We strictly followed the PRISMA extension statement for reporting systematic reviews incorporating NMA of health care interventions (PRISMA-NMA).^[10]^ This NMA protocol has been registered in the PROSPERO platform (CRD42023462289). Ethical approval is not required because the information used in this study is obtained from published randomized controlled trials (RCTs).

### 2.1. Inclusion criteria

#### 2.1.1. Types of studies RCTs

Randomized controlled crossover trials only included pre-crossover data. Only studies in Chinese and English will be included, but not limited to country and publication status.

#### 2.1.2. Types of participants

Patients who met the 4th National Conference on Cerebrovascular Disease^[11]^ or other relevant diagnostic criteria were diagnosed with PSA through the aphasia screening tool.

#### 2.1.3. Types of intervention and comparators

The interventions in the experimental group included MNT, MT, SST, CILT, MIT, AT, rTMS, and tDCS, and the interventions in the control group were routine speech training, nursing care, and sham stimulation (RT), or rehabilitation interventions different from those in the experimental group.

#### 2.1.4. Types of outcome measures

The aphasia quotient (AQ) was used to assess the severity of the patient’s aphasia. The patient’s spontaneous speech, listening comprehension, repetition, and naming abilities were evaluated based on assessment scales such as the Western Aphasia Battery, the Aachen Aphasia Test, and the Boston Diagnostic Aphasia Test.

### 2.2. Exclusion criteria

1. Non-RCTs, such as reviews, guidelines, conference summaries, cell or animal experiments.
2. Interventions in the experimental group included treatment options other than the above or combined with more than 2 rehabilitation interventions.
3. Duplicated articles.
4. None of the above outcome indicators.
5. The resulting data were incomplete, could not be converted, or there were errors.

### 2.3. Literature search strategies

We searched 6 English databases: “PubMed,” “Cochrane,” “Web of Science,” “OVID,” “CINAHL,” and “Embase,” and 4 Chinese databases: “CNKI,” “WanFang,” “CBM,” and “VIP,” from the establishment of the database to August 28, 2023. The following search terms were used to search in the English database: “stroke,” “apoplexy,” “aphasia,” “post-stroke aphasia,” “Schuell stimulation,” “music therapy,” “melodic intonation therapy,” “constraint-induced language therapy,” “motor imagery therapy,” “mirror neuron,” “virtual reality,” “attention training,” “repetitive transcranial magnetic stimulation,” “transcranial direct current stimulation.” Equivalent search terms were used for the Chinese databases. At the same time, we used a combination of Medical Subject Headings and free-text search terms to adjust the retrieval strategy based on the retrieval characteristics of each database.

### 2.4. Literature screening and data extraction

After completing the preliminary literature search, we imported the obtained literature into the EndNote X9 software for management. Literature data for inclusion in the final decision will be extracted using a pre-developed table. Extracting information includes the following: first author, publication time, sample size, age, intervention, treatment time, and outcome indicators.

Two researchers conducted the above process independently, with the cross-checked; in case of disagreement, a discussion was required. If consensus could not be reached, third-party consultation was possible.

### 2.5. Literature quality assessment

Two reviewers independently assess the risk of bias according to The Risk of Bias 2 tool for randomized trials in the Cochrane Handbook, based on the following domains:1. randomization process; 2. deviations from intended interventions; 3. missing outcome data; 4. measurement of the outcome; 5. selection of the reported result. Furthermore, each item is classified into 3 levels: “low risk,” “high risk,” and “some concerns.”^[12]^ Any inconsistencies in the assessment results will be resolved through discussion with a third party.

### 2.6. Statistical analysis

Stata 17.0 software was used for data analysis. The outcome indicators in this study were all continuous variables; the standardized mean difference and 95% confidence interval were chosen as effect indicators. First, a network group command was used to preprocess the data and draw a network relationship diagram for each outcome indicator. If there was a closed loop, an overall inconsistency test was required, and when *P* > .05, a consistency model was used for data analysis; at the same time, the node-splitting method was used to assess the degree of consistency between the results of the direct and indirect comparisons. Furthermore, plotting the surface under the cumulative ranking curve (SUCRA) for various interventions, the higher the SUCRA value, the better the ranking. When the number of documents included in the analysis for an intervention is <3, and the SUCRA value is too high, a sensitivity analysis is performed after excluding the intervention to judge the stability of the results. Finally, comparison-corrected inverted funnel plots were used to test for publication bias and small sample effects.

## 3. Results

### 3.1. Literature search results

**Figure 1.**
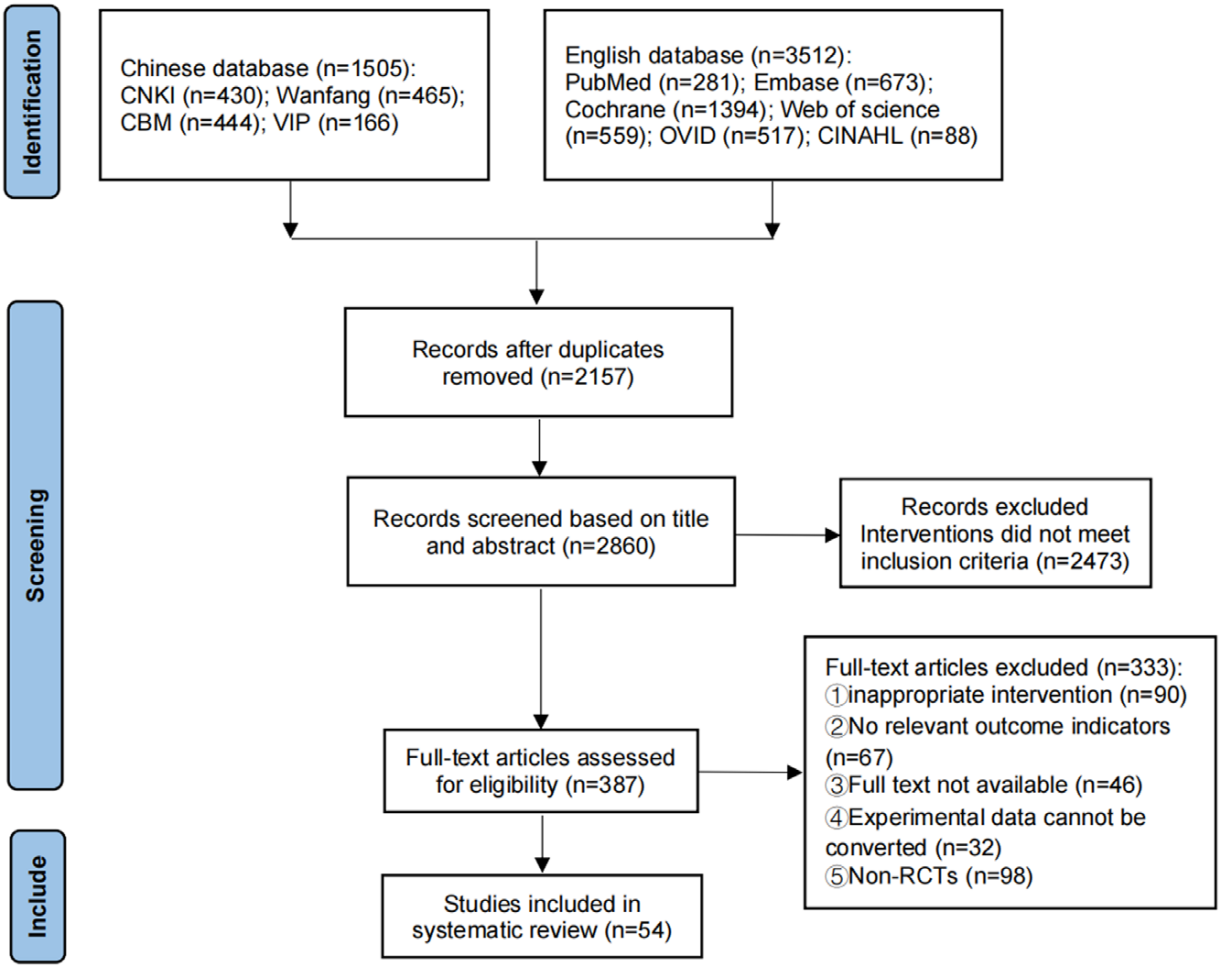
Flow chart of literature screening.

The initial search yielded 5017 publications. After removing duplicates, reading titles and abstracts, and reading the complete text, 54 studies were ultimately included.^[7,8,13–64]^ The literature screening process is shown in Figure 1.

### 3.2. Basic characteristics of the included studies

Of the 54 studies included, 6 were three-arm studies, 1 was a four-arm study, and the remaining 47 were two-arm studies. A total of 2688 PSA patients were included. The basic information of the literature is shown in Table 1.

**Table 1:** Basic information on literature.

Study	Sample size (cases)		Average age (year)		Rehabilitation interventions	Intervention time	Outcome indicators
	E	C	E	C			
Lin 2022^[13]^	17	16	54.71 ± 12.03	62.94 ± 14.59	rTMS	5d/w, 2w	③④⑤
Yuan 2020^[14]^	30	30	66.32 ± 9.54	69.13 ± 10.31	MNT	5d/w, 8w	③④
Woldag 2017^[15]^	20	20	71.3 ± 7.2	63 ± 14.3	CILT	5d/w, 2w	③④⑤
	20		70.3 ± 11.2				
Sickert 2014^[16]^	50	50	60.4 ± 11.9		CILT	2h, 15d	②③④⑤
Sun 2019^[17]^	20	20	52.2 ± 15.98	52.0 ± 12.1	MT-SST	5d/w, 12w	①②③④⑤
Chen 2020^[18]^	17	17	55.88 ± 11.76	62.18 ± 11.64	MNT	5d/w, 2w	①②③④⑤
Tan 2017^[19]^	34	34	49.96 ± 10.14	50.12 ± 11.60	MT-SST	5d/w, 8w	①②③④⑤
Zhang 2021^[20]^	20	20	52.90 ± 9.08	54.05 ± 10.81	MT	5d/w, 8w	①②③④⑤
Polanowska 2013^[21]^	18	19	57.6 ± 9.6	62 ± 11.9	tDCS	1t/d, 15d	③④⑤
Zhou 2023^[22]^	20	20	52.86 ± 11.75	54.72 ± 8.54	MT	6d/w, 4w	②③④⑤
Tao 2019^[23]^	16	15	51.31 ± 14.07	43.53 ± 9.44	tDCS	5t/w, 2w	①②③④⑤
Xie 2014^[24]^	15	15	63.13 ± 5.66	59.17 ± 6.04	CILT	5d/w, 2w	②③④⑤
Ciccone 2016^[25]^	9	8	69.4 ± 15.0	72.6 ± 14.1	CILT	20t, 4~5w	①
Li 2014^[26]^	30	30	NR		MIT	1t/d, 10d	③④
Zhou 2014^[27]^	10	10	53.3 ± 13.3	52.6 ± 11.2	CILT-rTMS	1t/d, 10d	②③④⑤
	10	10	55.2 ± 13.8	51.1 ± 12.2			
Dou 2021^[28]^	43	43	59.15 ± 5.04	58.71 ± 5.04	MIT	2t/d, 3w	③④
Fan 2017^[29]^	25	25	NR		rTMS	5d/w, 4w	①
Lin 2023^[30]^	16	17	54.06 ± 12.12	62.24 ± 14.42	rTMS	5d/w, 2w	③④⑤
Wu 2019^[31]^	40	38	55.2 ± 0.4	52.3 ± 0.6	MIT	2t/d, 2w	①②③④⑤
Fu 2018^[32]^	20	20	49.4 ± 8.5	57.4 ± 6.2	AT	1t/d, 4w	①②③④⑤
Zhang H 2019^[33]^	20	20	52.0 ± 10.17	54.95 ± 11.81	AT	6d/w, 5w	①②③④⑤
Cai 2016^[34]^	31	33	60.84 ± 16.48		MT	5d/w, 8w	③④
Chen 2021^[35]^	15	15	53.35 ± 11.56	55.75 ± 11.47	MNT	5d/w, 2w	①②③④⑤
Seniów 2013^[36]^	20	20	61.8 ± 11.8	59.7 ± 10.7	rTMS	5d/w, 3w	③④⑤
Tian 2017^[37]^	15	15	59.3 ± 12.4	62.1 ± 10.7	MNT	5d/w, 4w	①②③④⑤
Pei 2015^[38]^	30	30	54.19 ± 15.87	51.73 ± 13.30	AT	5d/w, 4w	①②③④⑤
Qu 2020^[39]^	40	40	65.1 ± 8.1	63.5 ± 10.4	MIT	2t/d, 35d	②
Zhu 2021^[40]^	10	10	60.4 ± 8.3	61.4 ± 12.0	MNT	6d/w, 3w	①②③④⑤
	10		61.6 ± 14.7				
Yoon 2015^[41]^	10	10	60.46 ± 9.63	61.13 ± 8.72	rTMS	5d/w, 4w	①②③④⑤
Bai 2022^[42]^	30	30	63.47 ± 7.81	59.91 ± 8.58	rTMS	5d/w, 4w	①②③④⑤
Wang L 2018^[43]^	21	21	54 ± 11.524	53.14 ± 10.641	tDCS	5t/w, 3w	①
Yang 2022^[44]^	20	20	76.4 ± 5.2	78.2 ± 5.3	tDCS	6d/w, 6w	②③④⑤
Tsai 2014^[45]^	33	23	62.3 ± 12.1	62.8 ± 14.5	rTMS	1t/d, 10d	④
You 2019^[8]^	21	21	56.52 ± 12.29	58.71 ± 12.00	MNT	5d/w, 4w	②③④⑤
Liu 2019^[46]^	45	45	64.5 ± 5.8	65.0 ± 5.4	MT	5d/w, 8w	①②③④⑤
Cui 2019^[47]^	36	36	43.29 ± 1.27	44.23 ± 1.31	rTMS	5d/w, 8w	①②③④⑤
Van 2016^[48]^	10	7	58.1 ± 15.2	63.6 ± 12.7	MT	5h/w, 6w	③④⑤
Gu 2019^[49]^	50	50	65.69 ± 7.21	67.30 ± 6.51	rTMS	5d/w, 4w	①
Haghighi 2018^[50]^	6	6	NR		rTMS	5d/w, 2w	①②③④⑤
Fang 2022^[51]^	26	26	53.2 ± 7.0	53.7 ± 7.2	CILT-tDCS	4w	①②③④⑤
	26		53.2 ± 7.6				
Ye 2021^[52]^	30	30	55.30 ± 6.69	54.63 ± 8.94	MT	5d/w, 4w	③④⑤
Lv 2023^[53]^	48	48	51.24 ± 8.48	50.75 ± 8.11	rTMS-MT	5d/w, 12w	①
	48	48	50.16 ± 7.92	51.98 ± 8.75			
Lee 2022^[54]^	13	13	56.61 ± 12.66	62 ± 14.4	rTMS	5d/w, 2w	③④
Li 2018^[55]^	15	15	65.3 ± 5.6	68.3 ± 5.8	rTMS	5d/w, 3w	①②③④⑤
Heiss 2013^[56]^	15	14	68.5 ± 8.19	69.0 ± 6.33	rTMS	12t	③④⑤
Zheng 2022^[7]^	15	15	53.27 ± 14.83	50.67 ± 16.50	AT-rTMS	5d/w, 4w	③⑤
	15		50.53 ± 13.85				
Zhao 2021^[57]^	8	10	58.00 ± 8.718	54.40 ± 9.395	tDCS	5d/w, 4w	①②③④⑤
Zhou 2021^[58]^	53	53	61.25 ± 8.41	59.87 ± 7.64	rTMS	5d/w, 4w	①②③④⑤
Haro 2018^[59]^	14	6	65.2 ± 15.1	61.7 ± 13.3	MT	12t, 6w	③④
Zhang SR 2019^[60]^	10	10	65.55 ± 4.49	65.72 ± 4.61	AT	1t/d, 8w	①②③④⑤
Wang 2020^[61]^	19	18	53.58 ± 10.59	50.50 ± 14.69	MT	5d/w, 4w	①②③④⑤
Rubi 2015^[62]^	15	15	67.9 ± 8.12	69.60 ± 6.67	rTMS	5d/w, 2w	③④⑤
Shi 2006^[63]^	10	10	51.7 ± 9.8		CILT	30–35 h, 2 w	②③④⑤
Wang CY 2018^[64]^	45	45	62.4 ± 8.4	62.0 ± 8.8	CILT	5d/w, 12w	①③

AT = attention training, C = control group, CILT = constraint-induced language therapy, d = day, E = experimental group, h = hour, MIT = motor imagery therapy, MNT = mirror neuron therapy, MT = music therapy, NR = not reported, rTMS = repetitive transcranial magnetic stimulation, SST = Schuell stimulation therapy, t = time, tDCS = transcranial direct current stimulation, w = week. ① = aphasia quotient, ② = spontaneous speech, ③ = listening comprehension, ④ = repetition, ⑤ = naming.

### 3.3. Literature quality assessment

**Figure 2.**
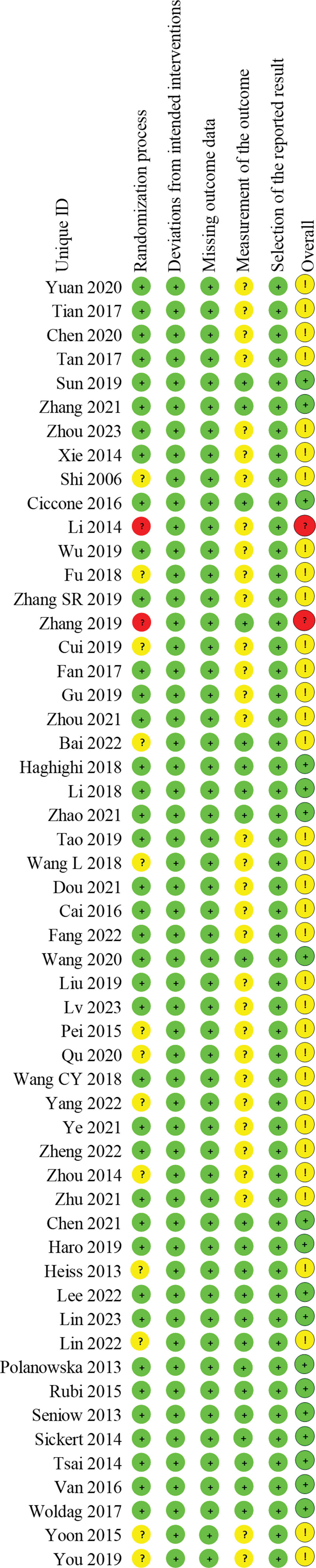
Summary of risk of bias.

**Figure 3.**
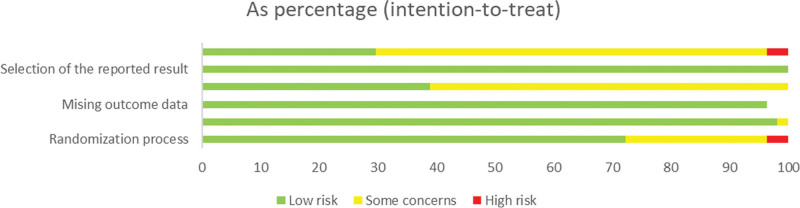
Risk of bias bar chart.

All 54 studies were RCTs, of which 36 used the random numbers table method, 2 studies randomized groups using opaque envelopes, 1 study performed stratified randomization, 1 study randomized groups using a coin toss, 1 grouped patients according to the odd or even of their tail number at the time of enrollment, and the remaining 13 did not mention the specific method of randomization; 11 studies used sealed envelopes for allocation concealment; 11 studies used a single-blind method for either the therapist or the assessor, and 10 studies used a double-blind method for 2 of the assessor, the therapist, and the patient; all studies had complete data. The results of the risk of bias evaluation are shown in Figures 2 and 3.

### 3.4. Results of NMA

#### 3.4.1. Evidence network relationships

**Figure 4.**
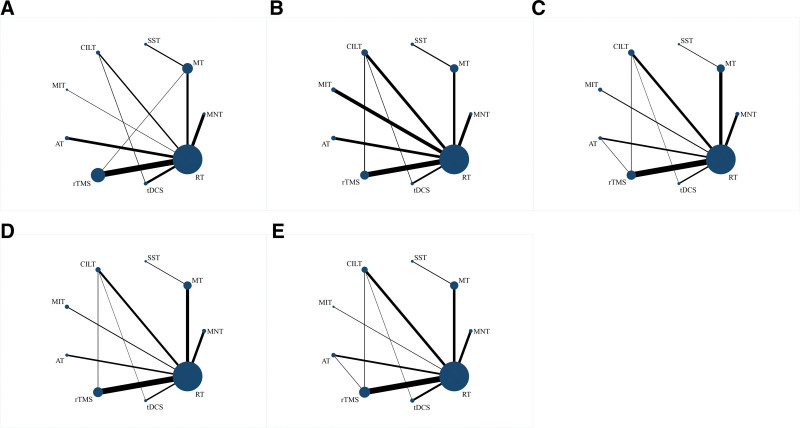
Network relationship diagram (A = aphasia quotient, B = spontaneous speech, C = listening comprehension, D = repetition, E = naming, RT = routine speech training, nursing care, and sham stimulation, SST = Schuell stimulation therapy, MNT = mirror neuron therapy, MT = music therapy, CILT = constraint-induced language therapy, MIT = motor imagery therapy, AT = attention training, rTMS = repetitive transcranial magnetic stimulation, tDCS = transcranial direct current stimulation).

The network of evidence for each intervention is shown in Figure 4. It can be seen that there are closed loops for each outcome indicator.

#### 3.4.2. Inconsistency check

The results of the overall inconsistency test showed that the *P* value of AQ score (*P* = .62), spontaneous speech score (*P* = .99), listening comprehension score (*P* = .76), repetition score (*P* = .17), and naming score (*P* = .93) were all > 0.05, so the consistency model was chosen for data analysis. The results of the local inconsistency test showed that all *P*-values were >.05, suggesting that each outcome indicator’s direct and indirect comparisons were relatively consistent. In addition, the results of the ring inconsistency test showed that the IF values for each outcome indicator ranged from 0.042 to 0.952, and the lower limit of the 95% CI contained 0, see Table 2.

**Table 2:** Loop inconsistency test results.

Outcome indicator	Loop	IF	*P*-value	95%CI	Loop-heterog-tau²
Aphasia quotient	CILT-tDCS-RT	0.067	.909	(0.00, 1.22)	0.076
	MT-rTMS-RT	0.072	.924	(0.00, 1.56)	0.333
Spontaneous speech	CILT-tDCS-RT	0.814	.072	(0.00, 1.70)	0.011
	CILT-rTMS-RT	0.395	.421	(0.00, 1.36)	0.001
Listening comprehension	CILT-rTMS-RT	0.344	.611	(0.00, 1.67)	0.117
	CILT-tDCS-RT	0.209	.725	(0.00, 1.37)	0.136
	AT-rTMS-RT	0.042	.944	(0.00, 1.23)	0.093
Repetition	CILT-tDCS-RT	0.952	.350	(0.00, 2.89)	0.448
	CILT-rTMS-RT	0.772	.248	(0.00, 2.08)	0.121
Naming	AT-rTMS-RT	0.420	.402	(0.00, 1.40)	0.059
	CILT-rTMS-RT	0.373	.643	(0.00, 1.95)	0.251
	CILT-tDCS-RT	0.109	.920	(0.00, 2.24)	0.617

AT = attention training, CILT = constraint-induced language therapy, MT = music therapy, RT = routine speech training, nursing care, and sham stimulation, rTMS = repetitive transcranial magnetic stimulation, tDCS = transcranial direct current stimulation.

#### 3.4.3. AQ score

**Figure 5.**
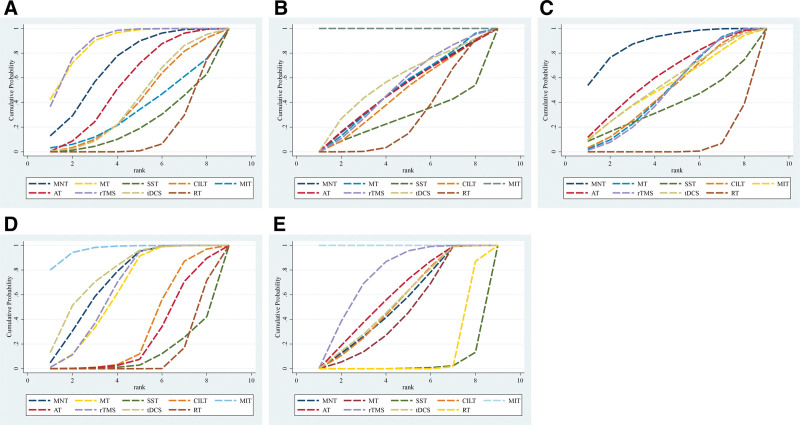
SUCRA probability ranking (A = aphasia quotient, B = spontaneous speech, C = listening comprehension, D = repetition, E = naming, MNT = mirror neuron therapy, MT = music therapy, SST = Schuell stimulation therapy, CILT = constraint-induced language therapy, MIT = motor imagery therapy, AT = attention training, rTMS = repetitive transcranial magnetic stimulation, tDCS = transcranial direct current stimulation, RT = routine speech training, nursing care, and sham stimulation).

A total of 29 studies reported changes in AQ scores. The results showed that the AQ scores of MNT, MT, AT, and rTMS were better than RT, and the AQ scores of MT were better than SST. The difference was statistically significant in all cases (*P* < .05), as shown in Table 3. The probability of SUCRA was ranked in the following order: rTMS (88.0) > MT (87.6) > MNT (70.3) > AT (55.3) > tDCS (41.0) > CILT (39.5) > MIT (32.6) > SST (21.7), see Figure 5A, Table 4.

**Table 3:** NMA of AQ scores.

Intervention	SMD (95%CI)								
	MNT	MT	SST	CILT	MIT	AT	rTMS	tDCS	RT
MNT	0								
MT	−0.26 (−1.01,0.49)	0							
SST	0.73 (−0.32,1.78)	**0.99 (0.26,1.72**)	0						
CILT	0.43 (−0.43,1.30)	0.69 (−0.11,1.50)	−0.30 (−1.38,0.79)	0					
MIT	0.56 (−0.56,1.67)	0.82 (−0.26,1.89)	−0.17 (−1.47,1.12)	0.12 (−1.03,1.27)	0				
AT	0.21 (−0.58,1.00)	0.47 (−0.26,1.19)	−0.52 (−1.55,0.50)	−0.23 (−1.07,0.61)	−0.35 (−1.45,0.75)	0			
rTMS	−0.25 (−0.92,0.42)	0.01 (−0.55,0.57)	−0.98 (−1.90, −0.06)	−0.69 (−1.42,0.04)	−0.81 (−1.83,0.21)	−0.46 (−1.10,0.18)	0		
tDCS	0.41 (−0.41,1.23)	0.67 (−0.09,1.43)	−0.32 (−1.37,0.73)	−0.02 (−0.73,0.69)	−0.14 (−1.27,0.98)	0.20 (−0.59,1.00)	0.66 (−0.02,1.34)	0	
RT	**0.76 (0.19,1.34**)	**1.02 (0.54,1.51**)	0.03 (−0.84,0.91)	0.33 (−0.31,0.97)	0.21 (-0.75,1.16)	**0.56 (0.02,1.10**)	**1.01 (0.67,1.36**)	0.35 (−0.23,0.94)	0

Bold values are statistically significant (*P* < 0.05).

AT = attention training, CILT = constraint-induced language therapy, MIT = motor imagery therapy, MNT = mirror neuron therapy, MT = music therapy, RT = routine speech training, nursing care, and sham stimulation, bolded text indicates *P*<0.05, rTMS = repetitive transcranial magnetic stimulation, SMD = standardized mean difference, SST = Schuell stimulation therapy, tDCS = transcranial direct current stimulation.

**Table 4:** Summary of SUCRA probability rankings.

Intervention	Aphasia quotient			Spontaneous speech			Listening comprehension			Repetition			Naming		
	No. of studies	SUCRA value	Ranking	No. of studies	SUCRA value	Ranking	No. of studies	SUCRA value	Ranking	No. of studies	SUCRA value	Ranking	No. of studies	SUCRA value	Ranking
MNT	4	70.3	3	4	48.6	5	6	88.1	1	6	70.9	3	5	52.0	6
MT	6	87.6	2	5	49.2	4	9	50.2	5	9	62.2	5	7	45.1	7
SST	2	21.7	8	2	26.1	8	2	37.5	8	2	10.6	8	2	2.1	8
CILT	3	39.5	6	5	44.6	7	7	49.5	6	6	32.0	6	6	53.0	5
MIT	1	32.6	7	2	100.0	1	3	53.2	4	3	96.5	1	1	100.0	1
AT	4	55.3	4	4	48.2	6	5	61.4	2	4	25.8	7	5	59.0	3
rTMS	9	88.0	1	7	50.5	3	14	49.2	7	14	64.2	4	13	73.7	2
tDCS	4	41.0	5	3	55.6	2	4	55.0	3	4	76.7	2	5	54.0	4

AT = attention training, CILT = constraint-induced language therapy, MIT = motor imagery therapy, MNT = mirror neuron therapy, MT = music therapy, rTMS = repetitive transcranial magnetic stimulation, SST = Schuell stimulation therapy, tDCS = transcranial direct current stimulation.

#### 3.4.4. Spontaneous speech

A total of 29 studies reported improvements in spontaneous speech ability. The results showed that the spontaneous speech scores of MIT were better than those of the remaining interventions, and the difference was statistically significant in all cases (*P* < .05), see Table 5. The order of probability of SUCRA was as follows: MIT (100.0) > tDCS (55.6) > rTMS (50.5) > MT (49.2) > MNT (48.6) > AT (48.2) > CILT (44.6) > SST (26.1), see Figure 5B, Table 4.

**Table 5:** NMA of spontaneous speech scores.

Intervention	SMD (95%CI)								
	MNT	MT	SST	CILT	MIT	AT	rTMS	tDCS	RT
MNT	0								
MT	0.05 (−2.80,2.90)	0							
SST	1.06 (−2.96,5.08)	1.01 (−1.83,3.85)	0						
CILT	0.17 (−2.55,2.89)	0.12 (−2.59,2.83)	−0.89 (−4.82,3.03)	0					
MIT	−**8.09** (−**11.98,** −**4.20**)	−**8.14** (−**12.03,**−**4.26**)	−**9.16** (−**13.97,**−**4.34**)	−**8.26** (−**12.05,**−**4.47**)	0				
AT	0.01 (−2.85,2.86)	−0.04 (−2.89,2.80)	−1.06 (−5.07,2.96)	−0.16 (−2.88,2.55)	**8.10 (4.21,11.99**)	0			
rTMS	−0.02 (−2.53,2.50)	−0.07 (−2.58,2.44)	−1.08 (−4.87,2.71)	−0.19 (−2.42,2.05)	**8.08 (4.43,11.73**)	−0.02 (−2.54,2.49)	0		
tDCS	−0.29 (−3.44,2.86)	−0.34 (−3.48,2.81)	−1.35 (−5.59,2.89)	−0.46 (−3.09,2.17)	**7.81 (3.69,11.92**)	−0.30 (−3.45,2.85)	−0.27 (−3.09,2.54)	0	
RT	0.63 (-1.39,2.65)	0.58 (−1.43,2.59)	−0.44 (−3.91,3.04)	0.46 (−1.36,2.28)	**8.72 (5.39,12.05**)	0.62 (−1.40,2.64)	0.64 (−0.86,2.15)	0.92 (−1.50,3.34)	0

Bold values are statistically significant (*P* < 0.05).

AT = attention training, CILT = constraint-induced language therapy, MIT = motor imagery therapy, MNT = mirror neuron therapy, MT = music therapy, RT = routine speech training, nursing care, and sham stimulation, bolded text indicates *P*<0.05, rTMS = repetitive transcranial magnetic stimulation, SMD = standardized mean difference, SST = Schuell stimulation therapy, tDCS = transcranial direct current stimulation.

MIT was excluded and reanalyzed due to its high SUCRA value and the small number of studies included in the analysis. The results showed that after excluding MIT, the results of direct and indirect comparisons were relatively consistent (*P* > .05), so the consistency model was used to analyze the data. The order of probability of SUCRA was as follows: tDCS (74.7) > rTMS (73.4) > MNT (73.2) > MT (63.3) > AT (59.6) > CILT (41.0) > SST (0.7), which did not change much from the previous results, suggesting that the results of the current study were relatively stable.

#### 3.4.5. Listening comprehension

A total of 46 studies reported improvement in listening comprehension ability. The results showed that the listening comprehension scores of MNT, MT, AT, and rTMS were better than those of RT, and the difference was statistically significant in all cases (*P* < .05), as shown in Table 6. The order of probability of SUCRA was as follows: MNT (88.1) > AT (61.4) > tDCS (55.0) > MIT (53.2) > MT (50.2) > CILT (49.5) > rTMS (49.2) > SST (37.5), see Figure 5C, Table 4.

**Table 6:** NMA of listening comprehension scores.

Intervention	SMD (95%CI)								
	MNT	MT	SST	CILT	MIT	AT	rTMS	tDCS	RT
MNT	0								
MT	0.45 (−0.23,1.13)	0							
SST	0.62 (−0.46,1.71)	0.17 (−0.67,1.01)	0						
CILT	0.45 (−0.25,1.15)	−0.00 (−0.65,0.64)	−0.17 (−1.23,0.89)	0					
MIT	0.41 (−0.45,1.27)	−0.04 (−0.85,0.77)	−0.21 (−1.38,0.95)	−0.04 (−0.86,0.79)	0				
AT	0.33 (−0.44,1.10)	−0.12 (−0.84,0.60)	−0.29 (−1.40,0.81)	−0.12 (−0.85,0.61)	−0.08 (−0.96,0.80)	0			
rTMS	0.45 (−0.17,1.07)	−0.00 (−0.56,0.56)	−0.17 (−1.18,0.84)	0.00 (−0.57,0.57)	0.04 (−0.72,0.80)	0.12 (−0.51,0.75)	0		
tDCS	0.40 (−0.43,1.23)	−0.05 (−0.83,0.73)	−0.22 (−1.37,0.92)	−0.05 (−0.77,0.66)	−0.01 (−0.95,0.92)	0.07 (−0.79,0.93)	−0.05 (−0.78,0.68)	0	
RT	**0.92 (0.40,1.44**)	**0.47 (0.03,0.91**)	0.30 (−0.65,1.25)	0.47 (−0.00,0.94)	0.51 (−0.17,1.19)	**0.59 (0.02,1.16**)	**0.47 (0.13,0.81**)	0.52 (−0.12,1.17)	0

Bold values are statistically significant (*P* < 0.05).

AT = attention training, CILT = constraint-induced language therapy, MIT = motor imagery therapy, MNT = mirror neuron therapy, MT = music therapy, RT = routine speech training, nursing care, and sham stimulation, bolded text indicates *P*<0.05, rTMS = repetitive transcranial magnetic stimulation, SMD = standardized mean difference, SST = Schuell stimulation therapy, tDCS = transcranial direct current stimulation.

#### 3.4.6. Repetition

A total of 45 studies reported improvement in repetition ability. The results showed that the repetition scores of MNT, MT, MIT, rTMS, and tDCS were better than those of RT and SST. MIT and tDCS were better than CILT. MIT was better than AT. The difference was statistically significant in all cases (*P* < .05), as shown in Table 7. The order of the probability of SUCRA was as follows: MIT (96.5) > tDCS (76.7) > MNT (70.9) > rTMS (64.2) > MT (62.2) > CILT (32.0) > AT (25.8) > SST (10.6), see Figure 5D, Table 4.

**Table 7:** NMA of repetition scores.

Intervention	SMD (95%CI)								
	MNT	MT	SST	CILT	MIT	AT	rTMS	tDCS	RT
MNT	0								
MT	0.11 (−0.41,0.64)	0							
SST	**0.91 (0.09,1.74**)	**0.80 (0.17,1.44**)	0						
CILT	0.51 (−0.06,1.08)	0.40 (−0.13,0.92)	−0.41 (−1.23,0.42)	0					
MIT	−0.45 (−1.09,0.19)	−0.57 (−1.16,0.03)	−**1.37 (-2.24,**−**0.50**)	−**0.96** (−**1.60,**−**0.33**)	0				
AT	0.61 (−0.02,1.23)	0.49 (−0.09,1.08)	−0.31 (−1.17,0.55)	0.10 (−0.52,0.72)	**1.06 (0.37,1.75**)	0			
rTMS	0.09 (−0.39,0.57)	−0.02 (−0.45,0.40)	−**0.83** (−**1.59,**−**0.06**)	−0.42 (−0.89,0.05)	0.54 (−0.02,1.10)	−0.52 (−1.06,0.02)	0		
tDCS	−0.07 (−0.73,0.58)	−0.19 (−0.80,0.43)	−**0.99 (-1.87,**−**0.10**)	−**0.58** (−**1.15,**−**0.01**)	0.38 (−0.34,1.10)	−0.68 (−1.38,0.02)	−0.16 (−0.74,0.42)	0	
RT	**0.81 (0.40,1.21**)	**0.69 (0.36,1.03**)	−0.11 (−0.83,0.61)	0.30 (−0.10,0.70)	**1.26 (0.76,1.75**)	0.20 (−0.28,0.67)	**0.72 (0.46,0.98**)	**0.88 (0.36,1.40**)	0

Bold values are statistically significant (*P* < 0.05).

AT = attention training, CILT = constraint-induced language therapy, MIT = motor imagery therapy, MNT = mirror neuron therapy, MT = music therapy, RT = routine speech training, nursing care, and sham stimulation, bolded text indicates *P*<0.05, rTMS = repetitive transcranial magnetic stimulation, SMD = standardized mean difference, SST = Schuell stimulation therapy, tDCS = transcranial direct current stimulation.

#### 3.4.7. Naming

A total of 40 studies reported improvements in naming ability. The results showed that the naming scores of MNT, MT, CILT, MIT, AT, rTMS, and tDCS were better than RT and SST. MIT was better than the rest of the rehabilitation interventions. The difference was statistically significant in all cases (*P* < .05), as shown in Table 8. The order of probability of SUCRA was as follows: MIT (100.0) > rTMS (73.7) > AT (59.0) > tDCS (54.0) > CILT (53.0) > MNT (52.0) > MT (45.1) > SST (2.1), see Figure 5E, Table 4.

**Table 8:** NMA of naming scores.

Intervention	SMD (95%CI)								
	MNT	MT	SST	CILT	MIT	AT	rTMS	tDCS	RT
MNT	0								
MT	0.08 (-0.56,0.71)	0							
SST	**1.07 (0.13,2.00**)	**0.99 (0.30,1.68**)	0						
CILT	−0.02 (−0.67,0.63)	−0.10 (−0.70,0.51)	−**1.09** (−**2.01,**−**0.17**)	0					
MIT	−**3.84** (−**5.10,**−**2.59**)	−**3.92** (−**5.15,**−**2.69**)	−**4.91** (−**6.32,**−**3.50**)	−**3.82** (−**5.06,-2.58**)	0				
AT	−0.08 (−0.75,0.59)	−0.16 (−0.78,0.47)	−**1.15** (−**2.08,**−**0.22**)	−0.06 (−0.70,0.58)	**3.76 (2.52,5.01**)	0			
rTMS	−0.23 (−0.79,0.34)	−0.30 (−0.81,0.20)	−**1.29** (−**2.15,**−**0.44**)	−0.21 (−0.72,0.31)	**3.62 (2.42,4.81**)	−0.15 (−0.67,0.38)	0		
tDCS	−0.03 (−0.71,0.66)	−0.10 (−0.74,0.54)	−**1.09** (−**2.03,**−**0.15**)	−0.00 (−0.58,0.57)	**3.82 (2.56,5.07**)	0.05 (−0.62,0.73)	0.20 (−0.36,0.77)	0	
RT	**0.62 (0.14,1.10**)	**0.54 (0.13,0.95**)	−0.45 (−1.25,0.35)	**0.64 (0.20,1.08**)	**4.46 (3.31,5.62**)	**0.70 (0.23,1.17**)	**0.85 (0.55,1.14**)	**0.64 (0.16,1.13**)	0

Bold values are statistically significant (*P* < 0.05).

AT = attention training, CILT = constraint-induced language therapy, MIT = motor imagery therapy, MNT = mirror neuron therapy, MT = music therapy, RT = routine speech training, nursing care, and sham stimulation, bolded text indicates *P*<0.05, rTMS = repetitive transcranial magnetic stimulation, SMD = standardized mean difference, SST = Schuell stimulation therapy, tDCS = transcranial direct current stimulation.

Sensitivity analyses were performed because only 1 MIT study was included, and its SUCRA value was too high. The results showed that the direct and indirect comparisons were relatively consistent (*P* > .05) after the exclusion of MIT, and therefore, a consistency model was used to analyze the data. The order of probability of SUCRA was as follows: rTMS (83.9) > AT (66.8) > tDCS (62.4) > CILT (61.4) > MNT (59.4) > MT (50.9) > SST (2.6), which was in the order of rTMS (83.9) > AT (66.8) > tDCS (62.4) > CILT (61.4) > MNT (59.4) > MT (50.9) > SST (2.6) and there was no change over the previous results, which suggests that the results of the current study were relatively stable.

#### 3.4.8. Publishing biased evaluations

**Figure 6.**
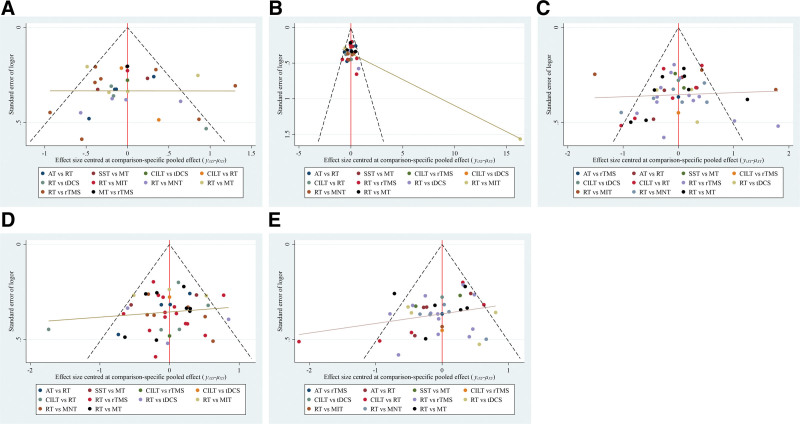
Comparison of outcome indicators—corrected inverted funnel plot (A = AQ score, B = spontaneous speech score, C = listening comprehension scoring, D = repetition score, E = naming score).

The results of the comparison-corrected inverted funnel plots showed that the scatters in each outcome indicator were unevenly distributed about the X = 0 vertical line, and some of the scatters were in the lower part of the funnel plots, suggesting that there might be a publication bias and a small-sample effect, see Figure 6.

## 4. Discussion

In recent years, there has been an increasing demand for speech therapy techniques, and researchers have been exploring and improving traditional speech training, resulting in a series of emerging speech rehabilitation interventions. However, the efficacy of different interventions for patients with PSA varies, and there is no conclusive evidence on which interventions are the most effective. Therefore, the present study systematically searched for 8 common speech rehabilitation interventions and included a total of 54 RCTs, which NMA systematically evaluated on 5 outcome indicators, namely, AQ scores, spontaneous speech, listening comprehension, repetition, and naming, and the degree of consistency of the results of direct and indirect comparisons of each outcome indicator was good.

Regarding improving AQ scores, rTMS was first in the SUCRA probability ranking, consistent with the results of the systematic evaluation by Zheng et al^[65]^ and the guideline recommendations.^[66]^ Interhemispheric inhibitory and compensatory models have been reported to be the primary theoretical basis for applying rTMS to PSA.^[67]^ rTMS induces a pulsed magnetic field perpendicular to the coil by stimulating the current in the coil, which generates tiny induced currents in the subcortex to modulate the membrane potential of neurons, inducing neural excitability in the functional areas of language to achieve the effect of inhibiting the mirror area of language in the nondominant hemisphere or promoting the functional area of language in the dominant hemisphere.^[68,69]^ Some studies have shown that the recovery of language function in PSA patients may be related to the altered functional connectivity of the interhemispheric language network.^[70]^ Moreover, rTMS can regulate the function of the dominant hemispheric language function area and the distal site, induce plastic changes in the functional connectivity between the hemispheres after stroke, and promote the reconstruction of the language network, thus improving the recovery of language function in PSA patients.^[71]^

In terms of improving listening comprehension ability, MNT was first in the SUCRA probability ranking, which is consistent with the results of previous traditional meta-analyses.^[72]^ The reason may be analyzed by the high degree of overlap between the parietal-frontal mirror system and the functional area of speech.^[73]^ Nishitani et al^[74]^ found that when volunteers were observing static pictures containing verbal and nonverbal lip shapes, their cerebral cortex was activated sequentially from the occipital lobe towards the superior temporal lobe, the inferior parietal lobe, and Broca area, in addition, when they imitated the pictures, the order of activation was the same as when they were observing. Furthermore, mirror neurons have been proposed as the basis for behavioral understanding, which translates visual information into cognition.^[75]^ The training system based on mirror neuron theory can produce visual and auditory stimulation and activate the nerve cells in the speech center by repeatedly playing videos of gestures and oral movements, thus improving the listening comprehension ability of PSA patients.^[35,76]^ Therefore, MNT may be the best rehabilitation intervention to improve the listening comprehension ability of PSA patients.

In improving spontaneous speech, retelling, and naming ability, MIT was the first in the SUCRA probability ranking. However, the number of MIT studies included in spontaneous speech and naming ability was minor, and the SUCRA value was too high, so we conducted sensitivity analyses. The results showed that the results of the SUCRA probability ranking before and after the exclusion of MIT were basically the same, suggesting that the results of the studies were stable. Motor imagery in PSA patients refers to the simulation of a series of articulatory actions, phrases, and sentences in the patient’s brain. This process can activate brain tissues in damaged language function areas, enhance sensory inputs, and promote the reconstruction of neural conduction pathways, thereby improving language function.^[77,78]^ In addition, MIT can be used as complementary or intensive training to other speech training performed at home after discharge from the hospital, which the patients easily accept. Qin et al^[79]^ showed that MIT was given based on conventional rehabilitation training, which can significantly improve the language comprehension and expression ability of PSA patients, consistent with this study’s results.

## 5. Limitations

This study still has some limitations: (1) the number of studies included in some of the interventions was minor, which may affect the reliability of the outcomes; (2) the results of the risk of bias evaluation showed that the overall quality of the included studies was not high, and some of the studies did not report the allocation of concealment and blinding, which posed some risk of bias; (3) there were differences in the type of aphasia of the patients, the duration of the treatment, the assessment criteria, and the method of assessment by the raters between studies, which may have led to the results of the evidence of a low quality.

## 6. Conclusion

In conclusion, rTMS was the most effective in improving aphasia severity in PSA patients, MNT was the most effective in improving listening comprehension ability, and MIT was the most effective in improving spontaneous speech, repetition, and naming ability. Because of the limitations of this study, clinical decision-makers need to be careful in formulating treatment plans according to the patient’s conditions to achieve the best therapeutic effects.

## Author contributions

**Conceptualization:** Congli Han, Jienuo Pan, Jinchao Du.

**Data curation:** Congli Han, Jienuo Pan, Luye Feng.

**Formal analysis:** Congli Han, Jinchao Du, Hengqin Ma.

**Methodology:** Congli Han, Jienuo Pan, Jinchao Du, Jiqin Tang.

**Software:** Congli Han, Jinchao Du, Luye Feng, Hengqin Ma.

**Validation:** Congli Han, Jiqin Tang.

**Writing – original draft:** Congli Han, Jiqin Tang.

**Writing – review & editing:** Congli Han, Jiqin Tang.
